# Clinical and Personal Predictors of Helmet-CPAP Use and Failure in Patients Firstly Admitted to Regular Medical Wards with COVID-19-Related Acute Respiratory Distress Syndrome (hCPAP-f Study)

**DOI:** 10.3390/biomedicines11010207

**Published:** 2023-01-13

**Authors:** Francesco Cei, Ludia Chiarugi, Simona Brancati, Silvia Dolenti, Maria Silvia Montini, Matteo Rosselli, Mario Filippelli, Chiara Ciacci, Irene Sellerio, Marco Maria Gucci, Giulia Vannini, Rinaldo Lavecchia, Loredana Staglianò, Daniele di Stefano, Tiziana Gurrera, Mario Romagnoli, Valentina Francolini, Francesca Dainelli, Grazia Panigada, Giancarlo Landini, Gianluigi Mazzoccoli, Roberto Tarquini

**Affiliations:** 1 Division of Internal Medicine I, San Giuseppe Hospital, 50053 Empoli, Italy; 2 Division of Internal Medicine II, San Giuseppe Hospital, 50053 Empoli, Italy; 3 Division of Internal Medicine, SS Cosma and Damiano Hospital, 51017 Pescia, Italy; 4 Division of Internal Medicine, Santa Maria Nuova Hospital, 50100 Firenze, Italy; 5 Division of Internal Medicine, Fondazione IRCCS Casa Sollievo della Sofferenza, 71013 San Giovanni Rotondo, Italy

**Keywords:** hCPAP, non-invasive ventilation, COVID-19, SARS-CoV-2, ARDS, prognosis

## Abstract

Acute Respiratory Distress Syndrome (ARDS) caused by COVID-19 is substantially different from ARDS caused by other diseases and its treatment is dissimilar and challenging. As many studies showed conflicting results regarding the use of Non-invasive ventilation in COVID-19-associated ARDS, no unquestionable indications by operational guidelines were reported. The aim of this study was to estimate the use and success rate of Helmet (h) Continuous Positive Airway Pressure (CPAP) in COVID-19-associated ARDS in medical regular wards patients and describe the predictive risk factors for its use and failure. In our monocentric retrospective observational study, we included patients admitted for COVID-19 in medical regular wards. hCPAP was delivered when supplemental conventional or high-flow nasal oxygen failed to achieve respiratory targets. The primary outcomes were hCPAP use and failure rate (including the need to use Bilevel (BL) PAP or oro-tracheal intubation (OTI) and death during ventilation). The secondary outcome was the rate of in-hospital death and OTI. We computed a score derived from the factors independently associated with hCPAP failure. Out of 701 patients admitted with COVID-19 symptoms, 295 were diagnosed with ARDS caused by COVID-19 and treated with hCPAP. Factors associated with the need for hCPAP use were the PaO_2_/FiO_2_ ratio < 270, IL-6 serum levels over 46 pg/mL, AST > 33 U/L, and LDH > 570 U/L; age > 78 years and neuropsychiatric conditions were associated with lower use of hCPAP. Failure of hCPAP occurred in 125 patients and was associated with male sex, polypharmacotherapy (at least three medications), platelet count < 180 × 10^9^/L, and PaO_2_/FiO_2_ ratio < 240. The computed hCPAP-f Score, ranging from 0 to 11.5 points, had an AUC of 0.74 in predicting hCPAP failure (significantly superior to Call Score), and 0.73 for the secondary outcome (non-inferior to IL-6 serum levels). In conclusion, hCPAP was widely used in patients with COVID-19 symptoms admitted to medical regular wards and developing ARDS, with a low OTI rate. A score computed combining male sex, multi-pharmacotherapy, low platelet count, and low PaO_2_/FiO_2_ was able to predict hCPAP failure in hospitalized patients with ARDS caused by COVID-19.

## 1. Introduction

Since the first wave of the Severe Acute Respiratory Syndrome Coronavirus 2 (SARS-CoV-2) pandemic, the main cause for hospitalization and the need for intensive care was the systematic development of Acute Respiratory Distress Syndrome (ARDS), with the need for oro-tracheal intubation (OTI) and high mortality [1,2].

Many differences were described between classical ARDS and Coronavirus Disease 19 (COVID-19)-associated ARDS. In particular, COVID-19 patients manifested severe hypoxemia, confirmed by arterial blood gases (ABG) analysis, without correspondent signs of respiratory distress; often, they did not feel dyspnea, so the term “happy hypoxemia” was widely used [3,4].

In fact, in the initial phases of COVID-19 pneumonia, the most common mechanisms of hypoxia were the alteration of the ventilation/perfusion matching, due to lung edema, alteration in lung perfusion regulation, and microthrombi formation in the lung [5,6,7], with the preservation of lung mechanics [8]; however, in late phases, lung mechanics often deteriorated in COVID-19 pneumonia. Differentiation into three phenotypes was proposed to individualize treatment: (1) Ground-glass opacities with good perfusion; (2) inhomogeneous atelectasis; and (3) a patchy ARDS-like pattern. Phenotype 1 required low positive end-expiratory pressure (PEEP) ventilation, while phenotype 2 needed higher PEEP values and phenotype 3 usually received OTI [9].

Categorical clinical practice guidelines are lacking, hence significant treatment variability was reported [10]. Widespread use of early intubation compared to noninvasive ventilation (NIV) was described; however, since the first wave, a progressive increase in steroid treatment and NIV was reported, with a reduction in mortality [11]. Many studies tried to find a difference between supplemental high-flow nasal cannula (HFNC) oxygen and NIV, with diversified results. The HENIVOT study failed to find a difference in the median number of days free of respiratory support within 28 days in patients with COVID-19 and moderate to severe hypoxemic respiratory [12]. The HELMET-COVID study did not find a statistically significant difference in 28-day all-cause mortality between helmet NIV and usual respiratory support (including conventional oxygen therapy, HFNC, and nose or/and face mask NIV) in adults with acute hypoxemic respiratory failure related to COVID-19 [13].

Our study aimed to estimate the use and success rate of helmet continuous positive airway pressure (hCPAP) and evaluate the factors associated with its delivery and failure in patients first admitted to a regular medical ward and developing COVID-19-related ARDS. We also aimed to derive a predictive score (hCPAP-f Score) to identify patients at admission at high risk for hCPAP failure in the context of ARDS caused by COVID-19.

## 2. Methods

### 2.1. Patients and Data Collection

We performed a monocentric retrospective observational study. We evaluated the charts of patients first admitted to general medicine wards (Division of Internal Medicine I and II of the San Giuseppe Hospital, Empoli, Italy) for COVID-19 symptoms between 6 March and 30 May 2020 and between 1 October 2020 and 15 March 2021. All admitted patients exhibited epidemiological, clinical, laboratory, and radiologic findings suggesting COVID-19. Diagnosis of SARS-CoV-2 infection was confirmed by a real-time polymerase chain reaction (RT-PCR) assay or a second-generation antigenic test performed on specimens collected by nasopharyngeal swab.

We included COVID-19 patients aged 18 years or older admitted to the emergency department for symptomatic SARS-CoV-2 infection (fever, cough, dyspnea, nausea and vomiting, diarrhea, thoracic pain, asthenia, myalgias, pharyngodynia, and loss of smell and taste).

We excluded patients first admitted to the Intensive Care Unit (ICU) and those admitted for other medical or surgical conditions with concomitant asymptomatic SARS-CoV-2 infection.

For all enrolled patients, we reported personal data including age, gender, comorbidities, day of symptoms onset, home treatments, and length of stay (LOS). Comorbidity definitions and home treatment specifications are reported in the Supplementary Materials (File S1: Specifications and definition). 

Clinical data, recorded at admission, included mean arterial blood pressure, the Glasgow Coma Scale (GCS), body temperature, cardiac frequency, peripheral oxygen saturation (SpO_2_), the ratio of oxygen saturation to the fraction of inspired oxygen [SpO_2_/FiO_2_ (S/F)], and the ratio of partial pressure of oxygen to the fraction of inspired oxygen [PaO_2_/FiO_2_ (P/F)].

Laboratory data, recorded at admission, included complete blood count (CBC); prothrombin time (PT) expressed as the international normalized ratio (INR); activated partial thromboplastin time (aPTT); D-dimer value; fibrinogen; transaminases; total bilirubin; lactate dehydrogenase (LDH); C-reactive protein (CRP); procalcitonin (PCT); interleukin-6 (Il-6); brain natriuretic peptide (BNP); arterial partial pressure of oxygen (PaO_2_) and carbon dioxide (PaCO_2_); and PaO_2_/FiO_2_ ratio (P/F).

Radiology findings acquired by computer tomography (CT) or conventional radiology scans included the presence of interstitial pneumonia.

ARDS was defined by the Berlin Criteria [14] evaluated at admission; criteria were as follows: Beginning of the symptoms in the last seven days or worsening in the last seven days; the presence of bilateral opacities confirmed by conventional radiology or CT; respiratory distress not supportively explained by cardiac failure or fluid overload; and a PaO_2_/FiO_2_ (P/F) ratio below 300. The severity of the disease was also evaluated with the CALL Score [15].

**Figure 1:**
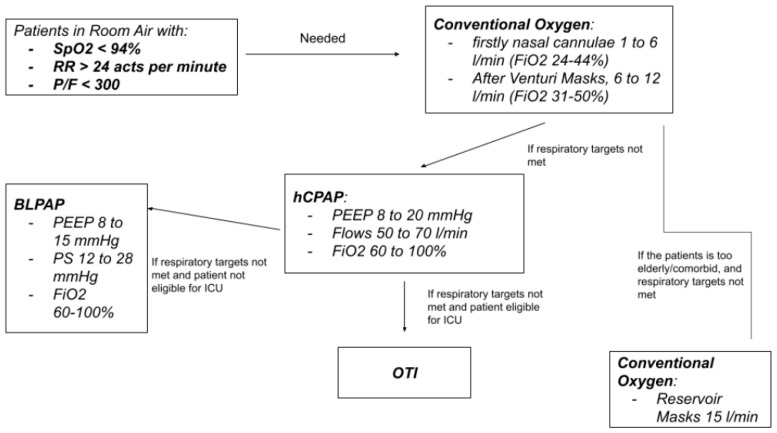
Stepwise approach to oxygen and ventilatory support for patients with COVID-19-related respiratory failure.

hCPAP was delivered as first-line noninvasive respiratory support in patients in whom conventional supplemental oxygen therapy delivered via a simple mask, a Venturi mask (VM), or a non-rebreather mask failed to achieve and maintain respiratory targets. In particular, hCPAP was delivered in pure hypoxemic respiratory failure when oxygen supply with VM at 50% FiO_2_ failed to maintain the target SpO_2_ (94–98%) and respiratory rate (RR < 24 acts per minute). We present the stepwise approach to oxygen and ventilatory support in Figure 1.

We delivered hCPAP using helmets that did not require a dedicated ventilator. These helmets convey high-flow medical gases in a closed space to generate the positive end-expiratory pressure (PEEP), required for alveolar recruitment.

High flows were generated by two systems:

– Flow-meters using both high-pressure oxygen and high-pressure medical air, with a target flow of 60 L/min at the beginning and a FiO_2_ of 60%, obtained by mixing the flows of air and pure oxygen; both air and pure oxygen could generate a flow of 60 L/min, with the theoretical possibility of attaining 100% FiO_2_;
– Flow meters using Venturi systems to generate the high flow; these systems convey oxygen in two ways with a maximum of 30 or 60 L/min to a strict canal in a Venturi valve; the high flow generates a low-pressure area, which recruits room air at high flows; this mix could generate an initial FiO2 of 60%, and upon closing the Venturi valve, we obtain a FiO_2_ of 100%.

hCPAP was set to deliver 50 to 70 L/min flow and at least 8 mmHg PEEP (titrated to 20 mmHg) for almost 12 h per day, divided into 3 cycles (morning, afternoon, and night) alternated to HFNC (using the first type of flow meters and set with at least the same FiO_2_ and flow) or non-rebreathing reservoir masks or Venturi masks (set with at least the same FiO_2_).

BiLevel positive airway pressure (BLPAP) was delivered as first-line respiratory support only when respiratory acidosis occurred.

When hCPAP failed to maintain the respiratory targets (SpO_2_ 94–98% and a RR < 24 acts per minute) despite titrating FiO_2_ to 80–100% and PEEP to 15–20 mmHg, we could consider two ways to increase the respiratory support:

– If the patient, evaluated by a trained intensivist, was considered recruitable for ICU, OTI was performed.
– If the patient, after collegial evaluation by the intensivist and the internist, was considered to have a scarce brief-term prognosis, was very elderly, and had multiple comorbidities, a trial for BLPAP was considered.

BLPAP was also delivered in the case of the appearance of moderate respiratory acidosis (pH 7.25–7.30). We usually started with pressure support of 12 mmHg (titratable to 26–28 mmHg) and PEEP of 8 mmHg (titratable to 15 mmHg), with at least the same FiO_2_ as in hCPAP.

Technical details of the devices used for hCPAP and BLPAP delivery in regular medical wards are reported in the Supplementary Materials.

BLPAP delivery and ICU admission decisions involved collaboration between internists and intensivists, but the decision to intubate the patient pertained to the intensivists.

We calculated the number of days from admission and from symptom onset to the beginning of hCPAP.

For patients who received hCPAP, we reported data on in-hospital pharmacological therapy, particularly the use of steroids (dexamethasone 8 mg or equivalent), venous thromboembolism prophylaxis (enoxaparin 4000 UI or equivalent), tocilizumab (intravenously 8 mg per kilogram of actual body weight, up to a maximum of 800 mg, in two infusions, 12 h apart, or subcutaneously at 162 mg administered in two simultaneous doses, one in each thigh, up to 324 mg in total), and antibiotics (beta-lactams, glycopeptides, aminoglycosides, tetracyclines, quinolones, and oxazolidinones).

The primary outcomes were hCPAP delivery and failure rates. hCPAP failure was a combined endpoint including the need for BLPAP or OTI as a rescue respiratory support technique and mortality during ventilation. The secondary outcome was the combination of intra-hospital death and the need for OTI.

We retrospectively collected patients’ data by reviewing paper and digital medical records (ARGOS version 4.2422820 and GALILEO version 1.5.3.14.2787 by Dedalus Italy S.p.A., via di Collodi 6/C, 50141 Florence, Italy). A structured web-based data collection form was developed for the retrospective chart review and for collecting clinical and personal data.

Data were collected by the physician staff of the Division of Internal Medicine I and II of the San Giuseppe Hospital, Empoli, Italy. Retrospective chart review studies relying on previously collected data may be wronged by biases due to the study operations, data collection, data entry, and data quality declaration causing a loss of information or approximation. To minimize this possibility, the first author comprehensively and carefully revised data collection, while also verifying the sources in the case of missing data, to curtail errors and biases. Data were analyzed after anonymization.

We included all the patients who met the inclusion criteria during the period described above. Regarding power and sample size calculation, designed for an observational study, the sample size was calculated considering differences between groups (hCPAP success and hCPAP failure); we considered a probable rate of hCPAP failure of approximately one-third [12,13] (ratio 2:1). Considering alpha 0.05 and power 0.90, and a Cohen’s effect size *d* of 0.4, we calculated a necessary sample size of at least 254 patients (169 with hCPAP success and 85 with hCPAP failure).

The study was carried out and is reported according to the Strengthening the Reporting of Observational studies in Epidemiology (STROBE) guidelines for observational studies [16].

The local Ethical Committee approved the study (BIGCOVID, No. 2161 date 6 September 2021).

Patients gave their written informed consent to participate. Only data collection from clinical records was allowed for patients unable to give their consent or those deceased. The study was conducted according to the Declaration of Helsinki for experiments involving humans.

### 2.2. Statistical Analysis

Continuous variables were reported as means and their 95th percentile confidence intervals (CIs) if normally distributed, and as medians and interquartile ranges (IQRs) if non-normally distributed. The D’Agostino–Pearson test of normality was used to test the normal distribution. Categorical variables were reported as absolute counts and percentages.

Differences in continuous variables between groups were tested with the *t*-test in normally distributed variables, with the Mann–Whitney test in non-normally distributed variables. Differences over time were tested with a paired-sample *t*-test or Wilcoxon test.

Categorical variables were tested with the Chi-square (χ^2^) probability distribution test and the Chi-square (χ^2^) test for trends (Cochran–Armitage test for trends).

We calculated Odds Ratios (ORs) and their 95th percentile CIs in univariate and multivariate logistic regression models. Only variables that resulted in being significantly different in the univariate analysis were included in the multivariate analysis. For continuous variables that resulted as statistically significant in the univariate analysis, we calculated ORs at values associated with the best of their sensitivity and specificity according to Youden’s J statistic (Youden index) for the primary outcomes [17] (see also Supplementary Materials).

We performed a retrospective database analysis, with some clinical and laboratory data eventually being corrupted, deleted, and/or made unreadable at random. We reported data and univariate analysis for all the variables included in the study. Listwise deletion of missing values was performed, and to maintain the power of the analysis, variables with a loss of data of over 10% were not included in the multivariate analysis, even if significantly altered in the univariate analysis.

We derivated a score (hCPAP-f Score) using the variables that resulted as independently associated with hCPAP failure; for categorical values, points were directly obtained by the OR in the regression models; for continuous variables, we estimated the ORs for each quartile of distribution to obtain correspondent points. An OR in the range between 0.5 and 1.5 was considered 0 (not influent).

We tested the ability of the derived score to predict hCPAP failure by calculating the area under the curve (AUC) of the receiver operating characteristic (ROC) curves and tested the non-inferiority with both the Call Score and IL-6 serum levels. We estimated both sensitivity and specificity. We also tested the score for the secondary outcomes.

For all analyses, a *p*-value below 0.05 was considered statistically significant.

All the statistical analyses were performed using MedCalc statistical software (MedCalc Software, Acacialaan 22, 8400 Ostend, Belgium). The sample size was calculated with G*Power (The G*Power Team, Heinrich-Heine-Universität Düsseldorf, Universitätsstr. 1 40225 Düsseldorf, Germany).

## 3. Results

**Figure 2:**
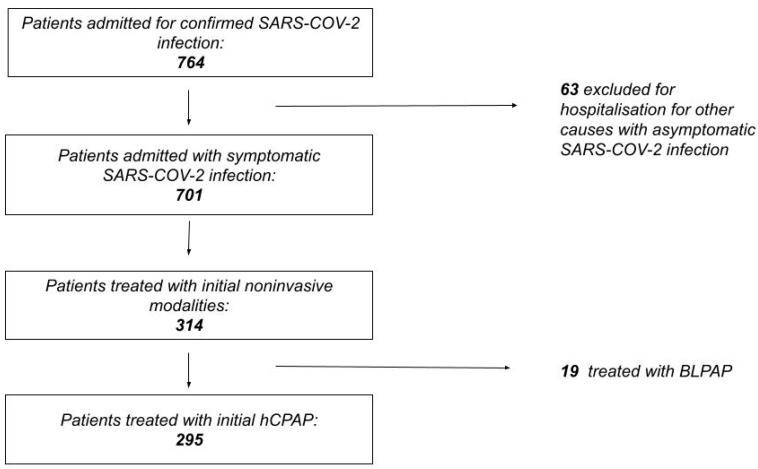
Flow diagram rendering the process of patient selection.

A total of 764 patients were admitted in the regular medical wards for COVID-19 respiratory symptoms, 63 (8.2%) were excluded for hospitalization due to other acute medical or surgical conditions, and 463 (66%) were admitted with clinical characteristics of ARDS, with a severe increase in the risk of OTI and death (OR 8.9, CI 4.8–16, *p* < 0.001). The overall rate of death and OTI was 23.5% (165), in-hospital mortality was 20.3% (142), and 36.6% (52) were in the non-ventilated group. The median length of stay in the hospital was 11 days (7–17) (Figure 2).

Noninvasive mechanical ventilatory support was needed in 314 patients (44.8% of the patients included in the analysis and 67.8% of patients with COVID-19-related ARDS). Furthermore, 19 patients (6.1%) needed BLPAP ab initio and 295 patients started a trial of hCPAP. Moreover, 86 patients (12.3%) were transferred to ICU, and OTI was needed in 46 patients (6.5%).

Of the patients treated with hCPAP, only three patients did not meet all of Berlin’s criteria for ARDS, for higher values of P/F at admission; however, they met Berlin’s criteria for ARDS during hospitalization. No patient without ARDS was treated with both BLPAP and OTI.

The median number of days from admission to the beginning of hCPAP was 2 (1–3), and between symptoms onset and the beginning of hCPAP, it was 7 (5–9). The median duration of noninvasive ventilation was 6 days (2–10) and the median length of stay in hospital for ventilated patients was 15 (11–24, *p* < 0.001). Supplemental HFNC oxygen alternated with hCPAP was delivered to 203 patients (68.8%).

Among the patients who needed noninvasive ventilation, 290 (92%) received steroids, 283 (90%) thromboprophylaxis (however, the other patients continued oral anticoagulation), 204 (64.9%) antibiotics, and 56 (17.8%) tocilizumab.

Differences between patients who needed hCPAP and those treated with conventional oxygen supplementation are reported in Table 1. Pre-existing factors associated with an increased risk of the need for hCPAP were the following: Young age, male sex, the presence of chronic kidney disease (CKD), active neoplasia, and severe obesity; lower applications of hCPAP were found to be associated with neuropsychiatric disorders, a serious risk factor for nonadherence and poor compliance. These patients also showed higher hemoglobin values, neutrophils count, INR, transaminases, fibrinogen, LDH, inflammatory markers, and Call Score values, in addition to lower P/F.

**Figure 3:**
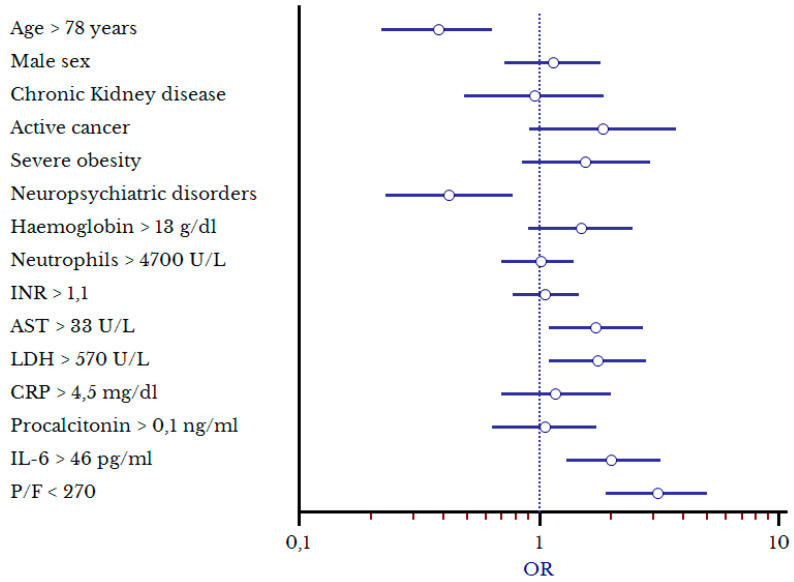
Forest Plot of the factors associated with increased risk of hCPAP delivery.

Independent factors associated with the need for hCPAP confirmed by multivariate analysis (Table 2, Figure 3) were the following: P/F < 270 (OR 3.1, 1.9–4.9), IL-6 serum levels > 46 pg/mL (OR 2, 1.3–3.2), AST > 33 U/L (OR 1.7, 1.1–2.8), and LDH > 570 U/L (OR 1.75, 1.1–2.8). Moreover, a reduction in the administration of hCPAP was found in patients 78 years old and older (OR 0.38, 0.23–0.64) and in those with neuropsychiatric disorders (OR 0.43, 0.23–0.78).

hCPAP failure occurred in 125 patients (42.4%); of them, 102 required OTI or died (34.5%), and 47 (15.9%) died during hCPAP without the advancement of respiratory support. In 25 patients, a trial of BLPAP was performed, and 11 (44%) died during BLPAP. After OTI, 23 (50%) patients died. Patients initially treated with BLPAP showed high rates of OTI and death (13.68%).

Patients with hCPAP failure were elderly, male, and affected by hypertension, cardiovascular diseases, respiratory diseases, CKD, active cancer, and neuropsychiatric disorders, polytherapy at home (at least three medications), and were treated with antibiotics during hospitalization. They also showed lower values of platelet count, fibrinogen, and P/F and higher values of total bilirubin, C-reactive protein, D-dimer, procalcitonin, interleukin-6, and serum creatinine. Details are shown in Table 3.

**Figure 4:**
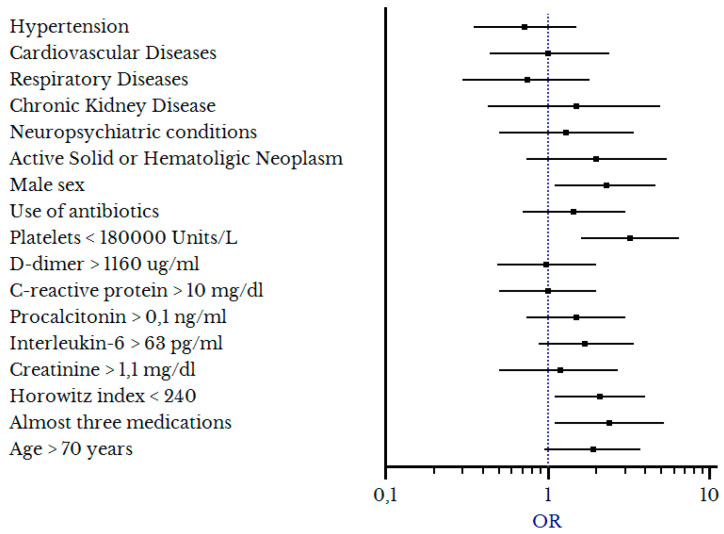
Forest plot rendering factors associated with hCPAP failure.

Multivariate regression models confirmed the following independent factors were associated with hCPAP failure: Male sex (OR 2.2, 1.1–4.8), polytherapy at home (at least three medications) (OR 2.4, 1.1–5.1), platelet count < 180 × 10^9^/L (OR 3.2, 1.6–6.2), and P/F < 240 (OR 2, 1.04–4), as detailed in Table 4 and Figure 4.

In Table 5, the derived hCPAP-f Score is presented, ranging from a minimum value of 0 to a maximum value of 11.5 (see Section 2).

**Figure 5:**
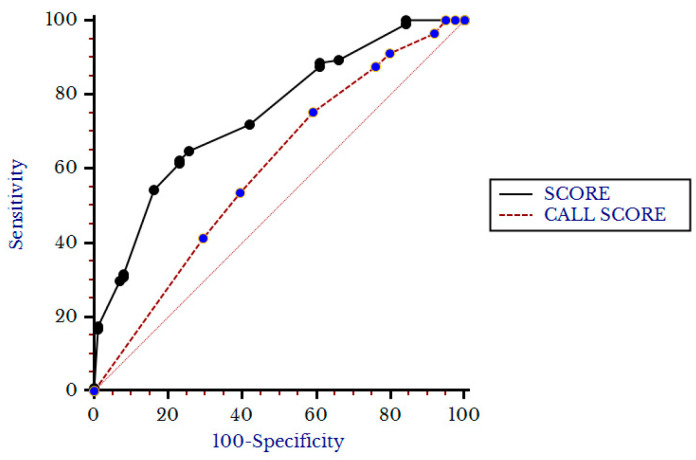
Comparison between Score and Call score for primary outcome.

In the group of patients forced to undergo hCPAP-f, the median hCPAP-f score value was 4.5 (2.5–6.5) and was higher in patients with hCPAP failure (6.5, 4–8.5) with respect to patients with hCPAP success (4, 2–5, *p* < 0.001). The hCPAP-f Score showed an AUC of 0.74 (0.69–0.79) and Younden’s value > 4.5, *p* < 0.001) with a sensitivity of 63% and a specificity of 74%. As shown in Figure 5, the hCPAP-f Score appeared superior to the Call Score in predicting hCPAP failure (AUC of Call Score 0.6, 0.53–0.66, *p* < 0.001).

**Figure 6:**
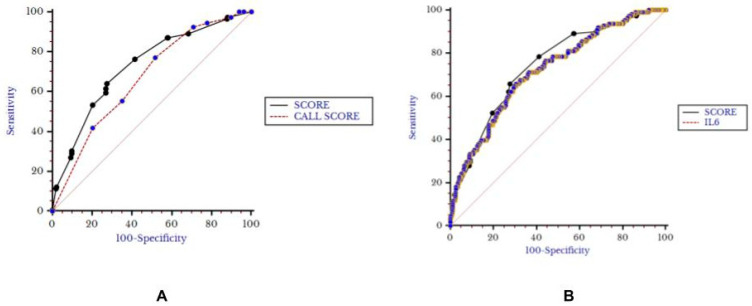
Comparison between Score and Call Score (**A**) and Interleukin-6 (**B**) for secondary outcome.

In the overall group of patients, the median hCPAP-f score value was also 4.5 (2.5–6.5), with higher values for patients who died or needed OTI (6.5, 4–8.5) with respect to other patients (4, 2–5, *p*< 0,001). The hCPAP-f score retained its predictive value for the secondary outcome (AUC 0.73, 0.69–0.76, Younden’s value > 4.5, *p* < 0.001, sensitivity 64% and specificity 72%) and appeared superior to the Call Score (AUC 0.67, 0.63–0.71, *p* = 0.037) and non-inferior to Interleukin-6 (0.72, 0.68–0.76, *p* = 0.58, Figure 6).

## 4. Discussion

During the SARS-CoV-2 pandemic, the prevalence of COVID-19-related ARDS in hospitalized patients varied substantially over time and geographical areas, and data in the scientific literature are heterogeneous. A report from the New York City area showed a prevalence of respiratory insufficiency of almost 27.8% and the need for intensive care of 14.2% with a high rate of OTI (12.2%) [18]. A register-based study of both the first and the second waves (March–December 2020) in Poland showed a very low prevalence of ARDS (3.6%) [19], while a higher percentage (32%) was found in Ethiopia during the second and third waves (September 2020–June 2021) [20]. In an early report from the Milan metropolitan area (February–March 2020), a very high prevalence (68%) of ARDS was found in hospitalized patients for COVID-19 [21]. A global literature survey reported an overall 26% prevalence of ARDS in hospitalized patients for COVID-19 [1]. Most of the studies included patients first admitted to both ordinary hospital and intensive-care beds. The heterogeneity of the prevalence of ARDS in the committed studies could be explained by many reasons, including the definition of ARDS used, the different rates of hospitalization in many countries, the inclusion of all patients with a positive RT-PCR or antigenic test (even those hospitalized for other reasons rather than symptomatic COVID-19), and the impact of virus variants.

In our experience, prior to the institution of mass vaccination programs, patients hospitalized for symptomatic COVID-19 had a high rate of ARDS after admission to medical regular wards (66%), with a median P/F of 242 (95–300) and a very significant increase in risk for adverse events (OR 8.9 for OTI and death). These could, in part, be explained by the ministerial recommendations for general practitioners to hospitalize patients with severe COVID-19 symptoms, particularly low oxygen desaturation index, dyspnea, and persistent fever. Moreover, inpatient transfers to the ICU were substantially low (12.5%) considering the disease severity detailed in Table 1.

The optimal respiratory support for COVID-19-related ARDS is not clearly defined. Experiences with early intubation lead to high mortality rates [2]. A choice between noninvasive modalities, including HFNC, CPAP, and BLPAP, was hazardous for the scarce data and the lack of indications from international guidelines. In Anglo-Saxon countries, an initial preference for HFNC was proposed [18,22]; this indication was based on data on undifferentiated acute hypoxemic respiratory failure, which showed no differences between conventional respiratory support, NIV, and HFNC in OTI rates, with a small advantage regarding 90-day survival for HFNC [23,24].

In our study, we reported data derived from our large experience in using hCPAP, frequently associated with HFNC, as a synergic oxygen delivery support technique. We report similar death and OTI rates to those reported by the recent Recovery-Rs prospective trial (34.3% in our study and 36.3% in the Recovery-Rs trial) [25].

Factors associated with an increased need for hCPAP were low P/F, increased interleukin-6 serum levels, increased AST values, and high LDH values. Our results are in agreement with those reported in a small study (97 patients) showing that fever > 37.5 °C, LDH value > 250 UI, and D-dimer over 1000 µg/mL were associated with the requirement for NIV [26]. In another small study considering all the noninvasive respiratory support techniques, only hypertension was found to be associated with an increased need for NIV [27].

In our study, transaminases, especially AST, were associated with an increased risk of death and OTI; AST is considered a marker of systemic disease, while ALT is considered a more liver-specific marker [28]. Increased LDH levels are also considered a marker of cell death/lisys and multi-organ failure due to cytokine storm, and it was associated with the severity of COVID-19 disease and the risk of death [29]. Interleukin-6 is a highly studied marker of immune activation in COVID-19, and its association with adverse outcomes (including the need for transfer to the ICU and death) was analyzed in systematic reviews [30]. The combination of these parameters, including low P/F, seemed to indicate that patients with unwarranted production of pro-inflammatory cytokines (cytokine storm) leading to ARDS and widespread tissue damage resulting in multi-organ failure are at increased risk of needing hCPAP delivery.

Despite being generally well tolerated, in our study, a reduction in the delivery of hCPAP was demonstrated in the elderly and in patients affected by neuropsychiatric disorders. However, as the decision to apply hCPAP was in the hands of the attending physicians, a very poor performance status, high dependency, and altered mentation could lead to more conservative and/or conventional approaches to oxygen therapy, using nasal prongs, cannula, or masks. Moreover, the benefit of ventilation in those patients could be questionable. In fact, the use of CPAP in the very elderly with acute hypoxemia due to heart failure did not lead to an increase in the survival of hospitalized patients [31], and the use of HFNC in very elderly patients with COVID-19 was associated with a high mortality rate (63.6%) [32].

Factors associated with hCPAP failure were male sex, polytherapy (the use of at least three medications at home), platelet count below 180 × 10^9^/L, and P/F < 240.

Male sex is associated with an increased risk of death and OTI in COVID-19 and other coronavirus syndromes, due to a different pattern of the immune response, considering that females had higher CD8+ and CD4+ lymphocyte counts, and stronger immunoglobulin response [33]. This was also confirmed by our study population, in which males had higher values of IL-6 with respect to females (*p* < 0.001). Furthermore, males were more frequently affected by almost one comorbidity, particularly hypertension and cardiovascular disease (*p* = 0.03) even if they were slightly younger (median age 70 vs. 73 years, *p* = 0.045). No differences were found in the risk of developing ARDS (*p* = 0.6).

No single comorbidity showed a significant association with hCPAP failure, while the use of multiple medications at home was associated with an increased risk of hCPAP failure, envisaging polytherapy as a marker of the patient’s frailty. Furthermore, the use of some medications (i.e., acetylsalicylic acid, digoxin, folic acid, mirtazapine, linagliptin, enalapril, atorvastatin, and allopurinol) was found associated with an increased risk of death [34]. COVID-19 can lead to thrombocytopenia via many mechanisms, including direct and direct platelet destruction, microthrombi formation, and reduced hematopoiesis induced by cytokine storm and low platelet count was also found associated with adverse events [35]. A very low P/F could suggest the need for more invasive treatments instead of hCPAP to resolve the hypoxemic respiratory failure. Furthermore, in an Italian cohort of COVID-19 patients hospitalized in 2020, the severity of ARDS (stratified as mild, moderate, and severe according to P/F value) was found to correlate with the risk of NIV failure (when considering both hCPAP and BLPAP) [36].

We derived a four-factor, 11.5-point score (hCPAP-f) showing good reliability in predicting hCPAP failure in COVID-19 patients. The hCPAP-f score was superior to the Call Score, first derived to detect the clinical deterioration of COVID-19 patients [15] and non-inferior for the secondary outcome in the overall COVID-19 patient population. It also resulted in being non-inferior to interleukin-6 serum levels, which we evaluated as a prognostic factor [37], to predict the secondary outcome in the overall COVID-19 patient population.

Varied results are found in the scientific literature regarding NIV failure prediction, and only the HACOR score was associated with CPAP failure, with an AUC of 0.74 [38]. However, in this observational study, many personal and laboratory factors analyzed in our study were not tested. A large multicenter study found low P/F, low platelet count, and high C-reactive protein to reliably predict NIV failure [39]. However, these two studies did not differ systematically between hCPAP and BLPAP, and both studies did not include the need for BiPAP in the outcomes as a rescue technique for patients who experienced hCPAP failure. Moreover, we also derived a new score to help physicians in rapidly stratifying high-risk patients for ICU transfer and selection for more aggressive respiratory support strategies (BLPAP, OTI).

Our study has some limitations, primarily related to its monocentric retrospective observational design, while the strengths are related to the real-world setting, the large sample size, and the great number of variables analyzed. Our derived score needs external validation for application in clinical practice.

## 5. Conclusions

Defining optimal respiratory support in COVID-19-related ARDS is challenging; nonetheless, NIV is realistically valuable to decrease the need for invasive mechanical ventilation, while the specific role of HFNC remains uncertain. As a first-line therapy, CPAP plus HFNC and additional interventions may be a useful option according to the patient’s condition and compliance [40].

In our clinical setting, hCPAP was largely used to treat COVID-19-related ARDS, leading to a low rate of OTI. Factors associated with the need for hCPAP were high AST, LDH, and IL-6 serum levels, as well as low P/F; older age and neuropsychiatric disorders led to a reduction in its use. Failure of hCPAP was associated with male sex, polytherapy with the use of at least three medications at home, low platelet count, and very low P/F. A feasible and reliable four-variable early score was derived, with good reliability in predicting both hCPAP failure and the combination of OTI and death in patients hospitalized for symptomatic COVID-19. The hCPAP-f score could be used at the time of hospital admission for the initial stratification of high-risk COVID-19 patients to assess the need for the selection of more aggressive respiratory support and/or early transfer to the ICU.

## Figures and Tables

**Table 1:** Differences between patients treated with hCPAP and conventional oxygen therapy.

Variables	hCPAP	Conventional Oxygen	Younden Index	*p*-Value
*Age (years)*	69 (61–78)	73 (58–84)	<78	0.006
*Male Sex n(%)*	189/295 (64%)	177/386 (45.9%)		0.01
*Hypertension*	142/295 (48.1%)	188/384 (48.9%)		0.93
*Cardiovascular disease*	80/295 (27.1%)	117/384 (30%)		0.14
*Respiratory diseases*	44/295 (14.9%)	60/384 (15.6%)		0.10
*Chronic kidney disease*	35/295 (11.8%)	68/386 (17.6%)		0.04
*Active cancer*	26/295 (8.8%)	28/386 (7.3%)		0.047
*Severe obesity*	42/295 (14.2%)	34/384 (8.9%)		0.004
*Neuropsychiatric disorders*	34/295 (11.5%)	99/386 (25.6%)		<0.001
*Diabetes*	50/295 (16.9%)	81/384 (21%)		0.08
*Polytherapy at home*	137/295 (46.4%)	183/384 (47.7%)		0.62
*Haemoglobin (g/dL)*	14 (13–15)	13 (12–15)	>13	<0.001
*Platelets (10^9^/L)*	207 (157–260)	204 (163–259)		0.98
*Neutrophils (units/L)*	5900 (4550–8600)	4900 (3370–7650)	>4700	<0.001
*Lymphocytes (units/L)*	800 (570–1100)	860 (600–1200)		0.30
*International Normalized Ratio (INR)*	1.2 (1.1–1.3)	1.1 (1.0–1.2)	>1.1	0.026
*D-dimer (µg/mL)*	847 (523–1550)	900 (530–1500)		0.95
*Fibrinogen (mg/dL)*	770 (640–880)	680 (570–790)		<0.001
*Aspartate aminotransferase (U/L)*	39 (31–54)	31 (24–46)	>33	<0.001
*Alanine aminotransferase (U/L)*	31 (19–51)	24 (16–39)		<0.001
*Lactate dehydrogenase (U/L)*	620 (480–760)	470 (380–600)	>570	<0.001
*Total Bilirubin (mg/dL)*	0.6 (0.5–0.8)	0.6 (0.5–0.8)		0.125
*C-reactive protein (mg/dL)*	8.3 (4–13)	4.8 (2–11)	>4.6	<0.001
*Procalcitonin (ng/mL)*	0.12 (0.07–0.3)	0.09 (0.05–0.22)	>0.1	<0.001
*Interleukin-6 (pg/mL)*	58 (31–100)	34 (14–66)	>46	<0.001
*Horowitz Index*	230 (130–275)	260 (180–310)	<270	0.006
*Brain natriuretic peptide (pg/mL)*	64 (35–140)	79 (34–190)		0.31
*Call score*	12 (10–13)	11 (9–12)	>9	<0.001

**Table 2:** Multivariate analysis of the factors associated with increased risk for need of hCPAP.

Variables	OR (CI)	*p*-Value
*Age > 78 years*	0.38 (0.23–0.64)	<0.001
*Male sex*	1.14 (0.72–1.81)	0.561
*Cronic kidney disease*	0.95 (0.49–1.86)	0.874
*Active cancer*	1.84 (0.91–3.73)	0.088
*Severe obesity*	1.57 (0.84–2.9)	0.153
*Neuropsychiatric disorders*	0.43 (0.23–0.78)	0.006
*Hemoglobin > 13 g/dL*	1.5 (0.91–2.46)	0.113
*Neutrophils > 4000 units/L*	1.02 (0.71–1.44)	0.933
*INR > 1.1*	1.06 (0.76–1.47)	0.72
*Aspartate aminotransferase U/L*	1.72 (1.08–2.74)	0.022
*Lactate dehydrogenase (U/L)*	1.75 (1.09–2.82)	0.021
*C-reactive protein > 5 mg/dL*	1.18 (0.71–1.96)	0.527
*Procalcitonin > 0.1 ng/mL*	1.06 (0.64–1.74)	0.829
*Interleukin-6 > 46 pg/mL*	2.04 (1.28–3.24)	0.003
*Horowitz index < 270*	3.11 (1.95–4.95)	<0.001

**Table 3:** Differences between patients with the success and failure of hCPAP. Younden indexes for hCPAP failure are also reported.

Variables	hCPAP Success	hCPAP Failure	Younden Index	*p*-Value
*Age (years)*	66 (57–73)	73 (63–83)	70	<0.001
*Male Sex*	98/170	94/125		0.002
*Hypertension*	74/170	17/125		0.037
*Cardiovascular disease*	38/170	44/125		0.021
*Respiratory diseases*	21/170	26/125		0.05
*Chronic kidney disease*	11/170	22/125		0.002
*Active cancer*	10/170	18/125		0.048
*Severe obesity*	26/170	16/125		0.53
*Neuropsychiatric disorders*	15/170	20/125		0.044
*Diabetes*	31/170	22/125		0.057
*Polytherapy at home*	62/170	77/125		<0.001
*Haemoglobin (g/dL)*	14 (13–15)	14 (13–15)		0.37
*Platelets (10^9^/L)*	220 (180–280)	174 (135–237)	<180	<0.001
*Neutrophils (units/L)*	6000 (4400–8500)	6000 (4400–8500)		0.59
*Lymphocytes (units/L)*	860 (600–1200)	780 (510–1040)		0.051
*International Normalized Ratio (INR)*	1.2 (1.1–1.3)	1.2 (1.1–1.3)		0.46
*D-dimer (µg/mL)*	730 (470–1280)	1070 (630–1900)	>1160	0.001
*Fibrinogen (mg/dL)*	800 (660–890)	720 (600–850)		0.001
*Aspartate aminotransferase (U/L)*	39 (25–60)	39 (25–60)		0.2
*Alanine aminotransferase (U/L)*	31 (20–53)	31 (20–53)		0.44
*Lactate dehydrogenase (U/L)*	620 (470–780)	620 (470–780)		0.11
*Total Bilirubin (mg/dL)*	0.6 (0.5–0.7)	0.7 (0.5–0.9)		0.027
*C-reactive protein (mg/dL)*	7.4 (3.7–13)	9.4 (5–14)	>7	0.013
*Procalcitonin (ng/mL)*	0.09 (0.06–0.2)	0.2 (0.1–0.43)	>0.1	<0.001
*Interleukin-6 (pg/mL)*	48 (24–84)	75 (40–130)	>63	<0.01
*Creatinine (mg/dL)*	0.87 (0.78–1.05)	1.07 (0.86–1.5)	>1.1	<0.001
*Horowitz Index*	250 (170–290)	190 (26–250)	<240	0.001
*Brain natriuretic peptide (pg/mL)*	58 (34–127)	75 (34–180)		0.16
*Call Score*	11 (9–13)	12 (10–13)	>10	0.002
*Days from hospital admission to hCPAP delivery*	2 (1–3)	1 (1–3)		0.58
*Days from symptom onset to hCPAP delivery*	8 (5–10)	7 (5–9)		0.083
*Days of ventilation*	8 (6–10)	7 (3–13)		0.233
*Therapy with tocilizumab*	25/170	26/125		0.10
*Therapy with antibiotics*	94/170	98/125		0.048

**Table 4:** Multivariate analysis for factors associated with hCPAP failure.

Variables	Odds Ratio (OR)	*p*-Value
*Age > 70 years*	1.86 (0.96–3.62)	0.066
*Male sex*	2.24 (1.12–4.78)	0.023
*Hypertension*	0.71 (0.35–1.44)	0.345
*Cardiovascular diseases*	1.01 (0.44–2.36)	0.973
*Respiratory diseases*	0.75 (0.31–1.81)	0.525
*Cronic kidney disease*	1.48 (0.44–4.98)	0.524
*Active cancer*	2.04 (0.75–5.5)	0.161
*Neuropsychiatric disorders*	1.26 (0.47–3.35)	0.646
*Polytherapy at home*	2.4 (1.09–5.13)	0.03
*Platelet count < 180 × 10^9^/L*	3.17 (1.58–6.34)	<0.001
*D-dimer > 1160 µg/mL*	1 (0.49–2.0)	0.979
*C-reactive protein > 10 mg/dL*	1 (0.5–2.01)	0.993
*Interleukin-6 > 63 pg/mL*	1.74 (0.89–2.45)	0.106
*Procalcitonin > 0.1 ng/mL*	1.47 (0.73–2.96)	0.281
*Creatinine > 1.1 mg/dL*	1.19 (0.53–2.67)	0.669
*Horowitz index < 240*	2.04 (1.04–3.99)	0.037
*Therapy with antibiotics*	1.47 (0.7–3.1)	0.31

**Table 5:** Odds Ratios (OR) and points used to compute the hCPAP-f score.

Variable	OR	Score Points
*Male Sex*	2.2	2
*Polytherapy at home*	2.4	2.5
*P/F* <84 84–240 241–300 >300	3.1 2.2 1.2 0.7	3 2 0 0
*Platelet count (× 10^9^/L)* <160 160–205 206–260 >260	3.9 2 1.3 0.85	4 2 0 0

## Data Availability

The data that support the findings of this study are available upon request from the first author, F.C. The data are not publicly available due to containing information that could compromise the privacy of research participants.

## Supplementary Materials

The following supporting information can be downloaded at: https://www.mdpi.com/article/10.3390/biomedicines11010207/s1, File S1: Specifications and definitions.
