# Supplementary material for: Clinical and Personal Predictors of Helmet-CPAP Use and Failure in Patients Firstly Admitted to Regular Medical Wards with COVID-19-Related Acute Respiratory Distress Syndrome (hCPAP-f Study)
Source: Biomedicines. 2023 Jan 13;11(1):207. doi: 10.3390/biomedicines11010207 (PMC9856077; doi:10.3390/biomedicines11010207)
Supplement: Supplementary file 1 [file biomedicines-11-00207-s001.zip › biomedicines-2059295-supplementary.pdf]

Supplementary Materials

Comorbidities definitions

Hypertension was defined by the 2018 ESC/EHRA Clinical practice guidelines for the management of Arterial Hypertension criteria.
Cardiovascular Diseases included Heart Failure, Chronic Coronary Diseases, Valvular Diseases, Atrial Fibrillation, Pulmonary Embolism, and Peripheral Arterial Disease as defined by the corresponding most recent ESC clinical practice guidelines criteria.
Diabetes was defined by the American Diabetes Association Standards of Medical Care 2022 Criteria.
Respiratory Diseases included Chronic Obstructive Pulmonary Disease (defined by the 2022 Global Initiative for Chronic Obstructive Pulmonary Diseases criteria), Asthma (defined by the 2022 Global Initiative for Asthma criteria), and Idiopathic Pulmonary Fibrosis (defined by the 2018 Diagnosis of Pulmonary Fibrosis an Official ATS/ERS/JRS/ALAT Clinical practice guidelines criteria).
Chronic Kidney Disease was defined by the 2012 KDIGO Clinical Practice Guidelines for Evaluation and Management of Chronic Kidney Disease criteria.
Solid or Hematologic neoplasm included all these conditions, as defined by the corresponding most recent National Comprehensive Cancer Network Guidelines criteria.
Severe Obesity was defined as a BMI over 35 kg/m^2, including both grade II and III of the 2015 European Guidelines for obesity management in adults.
Neuropsychiatric conditions include: previous ischemic stroke (defined by the 2021 AHA/ASA Guidelines for the Prevention of Stroke); previous spontaneous intracranial hemorrhages (defined by the 2022 Guideline for the Management of Patients With Spontaneous Intracerebral Hemorrhage: A Guideline From the American Heart Association/American Stroke Association); Dementia (defined by the 2018 Dementia: assessment, management, and support for people living with dementia and their carers NICE guideline); Parkinson's Disease (defined by the 2017 Parkinson's Disease in adults NICE guidelines); Epilepsy (defined by the 2014 International League Against Epilepsy Definition and Classification document); Major Depression, Anxiety Disorders needing pharmacological treatment, and Bipolar Disorders (defined by the Diagnostic and Statistical Manual of Mental Disorder criteria).

Pharmacotherapy specifications

Diuretics included thiazides, Henle loop diuretics, and mineralocorticoid receptor antagonists.
ACEI and ARBs also included sacubitril/valsartan.
Anticoagulants included both Vitamin-K antagonists and Direct Oral Anticoagulants.
Antiplatelets included low dosage acetylsalicylic acid and $P2Y_{12}$ ADP-receptor antagonists.
Antidiabetics included both insulin and oral and subcutaneous drugs (metformin, gliflozins, GLP1-inhibitors, DDP4-inhibitors, sulfonylureas, thiazolidinediones, meglitinides).
Neuroactive agents included benzodiazepines, tricyclic and non-tricyclic antidepressants (SSRI, SNRI), old and modern antiepileptics, typical and atypical antipsychotics, anticholinesterase, levodopa, and dopamine receptor agonists.

Devices used for hCPAP and Non-Invasive Ventilation

StarMed CaStar hood kit for CPAP use with Venturi flow generator and PEEP valve, medium, large and extra-large. Intersurgical S.p.A., Via Tonino Morandi, 12 - 41037 (MO), Italy. https://www.intersurgical.com/products/critical-care/starmed-castar-hood-for-cpap-therapy#downloads
Harol 9293-d-Cat Venturi flow generator. Harol Via Marcora 53, 20097 San Donato Milanese (MI), Italy.
Harol One Har CPAP helmet, medium, large and extra-large. Harol Via Marcora 53, 20097 San Donato Milanese (MI), Italy. https://harol.it/portfolio/casco-one-har/
Dimar CPAP helmet, medium, large and extra-large. Dimar S.r.L. Via G. Galilei, 6 41036 Medolla (MO) Italy https://www.crob.it/files/14/63/15/DOCUMENT_FILE_146315.pdf
The two helmets could be connected to both the flow-meters types, supporting the same flows and FiO2and generating the same PEEPs. The criteria for choice between them were based only on their availability, which varied during the pandemic waves.
Philps Respironics V60 mechanical ventilator. Philips S.p.A. , Viale Sarca 235, 20126 Milano, Italy.
Hamilton-C3 mechanical ventilator. Hamilton Medical AG, Bonaduz/Switzerland (Headquarters) Via Crusch 8, 7402 Bonaduz, Switzerland
Flow-meter variable air for oxygen RS series model double 15l/min-15l/min. Flow Meter Spa, Via del Lino, 6, 24040 Levate, Bergamo, Italy
Flow-meter RM series models 60l/min-60l/min and 30l/min-40l/min. Flow Meter Spa, Via del Lino, 6, 24040 Levate, Bergamo, Italy

Use of the Youden's Index

Using directly continuous variables in multiple regression models could lead to hard-to-interpret results (significant but with ORs of 1.001, 1.04, and similar), so values that could dichotomize populations led to more readable results. In a strongly affected population (as in our study), usual cut-off values could be unuseful, considering that substantially all the patients had altered parameters (CRP, leucocyte count, and more). Even using medians derived from the studied sample could lead to dichotomizing the population in a not useful manner. We reasoned that obtaining the Youden's Index, applicable for all the variables in the study independently of the normal ranges and medians, was the best way to obtain significant and easy-to-read results in multivariate models. Youden's Index is derived by the analysis of the Receiver Operating Characteristic (ROC) Curve for each variable and each outcome, founding the best value combining both sensibility and specificity, leading to optimal dichotomization. Even if the Youden's index is different in every population, it is however simple to replicate in further studies.
